# Eco-Friendly Polypropylene Composites Reinforced with Cellulose Fibers and Silica Nanoparticles

**DOI:** 10.3390/polym17101290

**Published:** 2025-05-08

**Authors:** Rinat M. Iskakov, Aigul S. Bukanova, Altynay S. Kalauova, Fazilat B. Kairliyeva, Alimzhan N. Nauashev, Gulbarshin K. Shambilova, Ivan M. Obidin, Mikhail S. Kuzin, Dmitryi N. Chernenko, Timofey D. Patsaev, Pavel S. Gerasimenko, Igor S. Makarov, Ivan Yu. Skvortsov

**Affiliations:** 1 Institute of Petrochemical Engineering and Ecology Named After N.K. Nadirov, Atyrau Oil and Gas University Named After S. Utebayev, M. Baimukhanov Street, 45A, Atyrau 060027, Kazakhstan; r.iskakov@aogu.edu.kz (R.M.I.); bukanova66@mail.ru (A.S.B.); kairlieva.fazi@mail.ru (F.B.K.); alimnau@gmail.com (A.N.N.); shambilova_gulba@mail.ru (G.K.S.); 2 Department of Chemical & Biochemical Engineering, Satbayev University, 13 Satbayev Street, Almaty 050013, Kazakhstan; 3 Department of Chemistry and Chemical Technology, Kh. Dosmukhamedov Atyrau University, Studenchesky Ave., 1, Atyrau 060011, Kazakhstan; skalauova@mail.ru; 4 A.V. Topchiev Institute of Petrochemical Synthesis Russian Academy of Sciences, Leninsky Prospekt, 29, Moscow 119991, Russia; vans1@bk.ru (I.M.O.); kuzms@ips.ac.ru (M.S.K.); onizsky@mail.ru (D.N.C.); gerasimenko11507@yandex.ru (P.S.G.); makarov@ips.ac.ru (I.S.M.); 5 National Research Center “Kurchatov Institute”, 1, Akademika Kurchatova Pl., Moscow 123182, Russia; timpatsaev@mail.ru

**Keywords:** cellulose, oligosiloxanes, polypropylene, composites, rheology, dynamic thermomechanical analysis, polycondensation, crystallinity, amorphization, sol–gel synthesis

## Abstract

In this study, polymer composites based on a polypropylene (PP) matrix with the addition of cellulose and ES-40, used as a silica precursor, were investigated. These composites were designed to achieve enhanced biodegradability through the incorporation of bioavailable cellulose and to enable subsequent carbonization into carbon–silicon carbide systems. Rheological investigations revealed that the multicomponent mixtures exhibited pseudoplastic behavior over the shear rate range typical of injection molding, ensuring process stability without additional plasticization. Morphological analysis demonstrated that an optimal balance of PP, cellulose, and ES-40 promoted the formation of a three-dimensional network structure, leading to a significant increase in flexural modulus at the equal flexural strength despite some reduction in tensile strength. It was further shown that substituting fibrous cellulose with microcrystalline cellulose improved the composite homogeneity, thereby enhancing the density and mechanical properties, especially in systems with low polymer contents. Preliminary pyrolysis experiments indicated that these injection-molded composites can serve as precursors for fabricating bulk thermally stable products containing silicon carbide particles. The obtained results underscore the high potential of the developed materials for applications in conventional injection molding, the possibility of additive manufacturing, and processes requiring subsequent carbonization.

## 1. Introduction

A wide range of polymer products are manufactured worldwide by the injection molding of melts, owing to its low cost, high productivity, and capability to produce various shapes [1,2]. Among the polymers employed for mass production, polypropylene (PP) is one of the most in demand due to its strength, ease of processing, and affordability [3]. To enhance the mechanical [4,5,6] and thermal [7,8,9] properties of PP products, numerous studies have focused on the development of composite materials [4,5,7,9,10,11,12,13,14,15]. Recently, the primary research direction for PP-based composites has been the formulation of compositions that preserve the mechanical properties of PP while exhibiting improved biodegradability [16,17].

One of the promising approaches in the development of biodegradable composites is the incorporation of natural components such as lignin [6,18] and cellulose [16,19]. Lignin is a by-product of the high-volume pulp and paper industry and is rarely processed [20]. At present, only exploratory studies are underway to identify promising pathways for its utilization and processing [21,22,23], with most research concentrating on its use as a dispersed phase in composite materials to enhance moduli of elasticity and biodegradation [24,25,26]. However, increasing the lignin content significantly diminishes the mechanical properties of the composites [27,28], which substantially limits the feasibility and range of potential applications. Therefore, natural fibers are of particular interest for the fabrication of highly efficient composites with enhanced biodegradability; owing to their anisotropy, they markedly improve the mechanical properties of the composites [14,15,17]. The most common reinforcing phase is cellulose fibers, obtained either from natural sources (cotton, miscanthus, flax) [20,29,30,31] or synthetic fibers produced by dry–wet spinning via lyocell [12,32,33,34,35] and viscose technologies [36,37,38]. Recently, microcrystalline cellulose (MCC) has gained wide acceptance due to its superior strength and rigidity [39] and excellent surface activity for binding [40], making it a promising filler for biocomposites.

Despite the excellent individual properties of both PP and cellulose, a major challenge lies in achieving their proper mixing and overcoming the low interfacial adhesion. This adhesion can be enhanced by the addition of maleic anhydride [41] or by incorporating rigid, anisotropic nanosized particles, such as silica [42,43]. The inclusion of rigid particles in composite materials not only improves the interfacial adhesion but also enhances the compressive mechanical properties of the material [44,45,46,47], which is particularly attractive for applications in outdoor furniture [48,49] and disposable tableware [50]. However, the introduction of a large number of rigid particles into a highly viscous melt, followed by processing at high deformation rates, poses significant challenges [51,52]. The process can be simplified by employing the sol–gel synthesis method for nanosized silica particles: at the mixing stage, the organosilicon compound exists as a low-viscosity liquid, which reduces the overall system viscosity [53], and after hydrolysis (facilitated by the residual moisture in cellulose), it solidifies [54], thereby enhancing both the interfacial adhesion and stiffness.

Of particular interest is the variation in the particle size distribution and the ratio between these rigid particles and cellulose fibers to control the mechanical properties of injection-molded products and to achieve a desired balance between compressive stiffness and tensile or flexural strength. Although a significant number of studies have focused on the influence of particle size distribution in powder injection-molding mixtures to maximize the degree of filling, the effect of the ratio of anisotropic fibers and rigid particles in injection-molded composite products has received little attention. Several studies have addressed the modeling of fiber orientation and distribution in injection-molded composites [55,56,57] as well as the impact of particle size on their mechanical and structural properties [58,59,60].

One of the key challenges in applying injection molding to produce precision parts is shrinkage [61,62]. In a recent study [63], it was demonstrated that the shrinkage of PP samples can be reduced by incorporating cellulose and silica, with the greatest effect achieved when hydrolytic polycondensation of the ethyl silicate is carried out during mixing.

Silica, in the presence of excess carbon, is reduced to form silicon carbide (SiC) at temperatures of approximately 1700 °C. This process, known as the Acheson process, is widely employed in industries for the synthesis of silicon carbide [64]. The introduction of carbon composites containing micro- and nanosized silica particles in an excess of carbon reduces both the processing temperature and time, thereby enabling the production of carbon–SiC composites with enhanced properties [65,66,67]. Examples of such materials include fibers based on composite PAN fibers with tetraethoxysilane [54] and cellulose fibers with vinyl triethoxyvinylsilane [68], where these compounds act as silica precursors.

However, the fabrication of bulk products from solutions using this method remains challenging. A promising solution is the combination of non-molten carbon precursors and silica precursors within a thermoplastic matrix. This approach opens up new opportunities for manufacturing bulk composite products that are applicable in both additive manufacturing and conventional injection molding.

The resulting material can be used either as a final product or as a “green” preform for the subsequent removal of the polymer binder, analogous to powder injection molding (PIM) technology [69,70,71,72]. In this process, the thermoplastic serves as a temporary binder, allowing the preform to be shaped, after which it is subjected to burnout and the subsequent sintering of the powder phase into a monolithic composite.

In this work, we investigated the use of ES-40 and cellulose as co-fillers in polypropylene-based composites suitable for both FDM and injection molding. We analyzed the role of the ES-40-to-cellulose ratio in the formation of a melt-processed hybrid structure capable of pyrolytic transformation into SiC. In contrast to prior formulations focused solely on mechanical reinforcement, our approach emphasized dual functionality: producing shaped “green” preforms that maintain their geometry during debinding and facilitate SiC formation, thereby offering a pathway to multifunctional composite systems.

## 2. Materials and Methods

### 2.1. Materials

Polypropylene (H030GP, Sibur, Moscow, Russia) was selected as the matrix to ensure the required flowability of the composition. Two types of cellulose were chosen as carbon-containing reinforcing fillers: wood fiber cellulose (Aditya Birla Group, Mumbai, India) with a moisture content of 5.6% and a degree of polymerization of 600 and microcrystalline cellulose (Farm Via, LLC., Moscow, Russia), which has a higher bulk density but a lower degree of polymerization, with particle sizes of 80–120 µm, a polymerization degree of 200, and a residual moisture content of 3%. Ethyl silicate 40 (ES-40) (Silane, Moscow, Russia) was used as a silicon-containing additive and served as a silica precursor.

**Figure 1:**
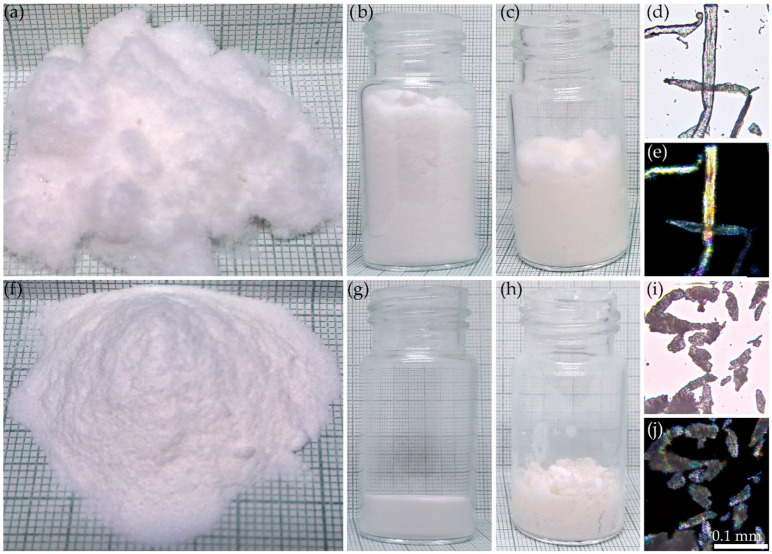
Images of fiber (**a**–**e**) and microcrystalline (**f**–**j**) cellulose samples with equal mass before (**a**,**b**,**f**,**g**) and after (**c**,**h**) impregnation with ES-40, as well as optical micrographs of the cellulose samples in transmitted (**d**,**i**) and cross-polarized transmitted (**e**,**j**) light.

Figure 1 presents images of the fiber (a–e) and microcrystalline (f–j) cellulose samples with identical masses before (a, b, f, g) and after (c, h) impregnation with ES-40, as well as optical microscopy images of the cellulose fibers and crystallites in transmitted (d, i) and cross-polarized transmitted (e, j) light.

The morphologies of the raw materials were substantially different. Fiber cellulose exhibits a bulk density five times lower than that of microcrystalline cellulose. Upon mixing with ES-40, the bulk density of the fiber cellulose increased, whereas the addition of ES-40 to the microcrystalline cellulose led to swelling, resulting in a decrease in its bulk density. Optical microscopy revealed that the fiber cellulose samples consisted of natural fibers with diameters of 20–40 µm and lengths ranging from 200 to 1000 µm, exhibiting pronounced orientation, as evidenced by intense birefringence in crossed polarizers. In contrast, the microcrystalline cellulose appeared as irregularly shaped particles with sizes varying from 5 to 150 µm.

### 2.2. Sample Preparation

Two- or three-component mixtures of PP, cellulose, and ES-40 were prepared by mechanical mixing in two stages. In the first stage, ES-40 was added to the cellulose, followed by mixing with a paddle mixer for 5 min until complete wetting of the cellulose was achieved. Subsequently, polypropylene granules were incorporated into the resulting pulp and mixed again to ensure their uniform distribution throughout the pulp volume. In the second stage, the mixing was carried out using a twin-screw laboratory micro-compounder, HAAKE Minilab II (Thermo Fisher Scientific, Dreiech, Germany), at 200 °C and 100 rpm for 10 min in a recirculating mode, followed by the injection of the molten material to obtain a composite extruded product.

The injection molding of the samples was performed using a pneumatic laboratory machine, IM12 (Xplore, Sittard, The Netherlands), with a nozzle (*d* = 4 mm) and a charging chamber temperature of 210 °C, a mold temperature of 70 °C, a maximum injection pressure of 0.55 MPa, and a pressure-holding time of 9 s. The steel molds used were designed in accordance with ISO 527-5:2009 [73] and ISO 604:2002 [74].

The component ratios in the investigated mixtures are listed in Table 1; for clarity and convenience, the concentration values have been rounded to whole numbers in the sample names.

Transparent films with thicknesses of 100 ± 20 µm were produced by hot-pressing the extrudate samples at 210 °C for the morphological analysis of the resulting composites by optical microscopy. For rheological studies and the fabrication of impellers, the extrudates were granulated.

### 2.3. Rheology

The rheological properties of the thermoplastic melt were studied using a Rosand RH-10 capillary rheometer (Malvern, UK). Flow curves were obtained at various shear rates ranging from 10 to 10^4^ s⁻^1^. A tungsten carbide capillary with a diameter of 500 µm and a length of 8 mm was used in all experiments. The flow curves were measured at a temperature of 200 °C, which is equivalent to the molding temperature.

### 2.4. Microscopy

The morphologies of the composite materials were examined on films with thicknesses of 100 ± 20 µm. Images were acquired using a “Biomed 6PO” microscope (Biomed, Moscow, Russia) equipped with a ToupTek E3ISPM5000 camera (ToupTek Photonics Co., Hangzhou, China) and a Plan-Apo objective with 4× magnification under transmitted and cross-polarized light.

### 2.5. Density Measurements

Density measurements were carried out using the hydrostatic weighing method under standard conditions in accordance with GOST 15139-69 [75], using 98% methanol as the working liquid. For each composition, at least 10 samples were studied, followed by standard statistical processing of the results with *p* = 0.05.

### 2.6. Mechanical Properties

The tensile properties of the samples were determined using an Instron 1122 testing machine (Instron, Norwood, MA, USA) at a crosshead speed of 10 mm·min^−1^ in accordance with ISO 527-5:2009 [74]. The flexural mechanical properties were measured by the three-point bending method, following GOST P 56810-2015 [76], ASTM D790 [77], and ISO 178 [78]. For each composition, at least 10 samples were studied, followed by standard statistical processing of the results with *p* = 0.05.

### 2.7. Pyrolysis

The carbonization of the samples was conducted in a Tammann-type electric graphite furnace with a power of 60 kW under an argon atmosphere with a volumetric flow rate of 0.5 m^3^·h^−1^ (under ambient pressure). The sample was placed in a cylindrical graphite crucible with a diameter of 30 mm and a height of 70 mm. The crucible was sealed with a graphite lid that featured a technological aperture to allow the escape of volatile carbonization products. The heating rate was set at 56.6 °C·min^−1^, and after reaching the target temperature of 1700 °C, an isothermal hold of 5 min was maintained. The temperature of the reaction volume was monitored through a furnace observation window by the radiation of the graphite heater using an infrared pyrometer, Kelvin Compact 3000 D (JSC “EUROMIX”, Moscow, Russia), with an accuracy of 0.2%.

### 2.8. Scanning Electron Microscopy (SEM)

The fiber morphology was analyzed using a Helios Nanolab 600 scanning electron microscope (SEM) (Thermo Fisher Scientific, Waltham, MA, USA) equipped with an AMETEK energy-dispersive X-ray spectrometer (EDAX, Pleasanton, CA, USA) and two secondary electron detectors: an ETD (Everhart–Thornley Detector) and a TLD (through-the-lens detector). Images were obtained at accelerating voltages of 2 kV and 10 kV and a current of 0.69 nA. The samples were mounted vertically onto carbon tape.

### 2.9. X-Ray Phase Analysis

X-ray phase analysis was performed on a Rigaku Rotaflex D/max-RC instrument using copper CuK α-radiation (λ = 0.154 nm). The diffraction pattern from the sample was registered in the angular range of 2θ = 10–120° with a step of 0.02° and a recording rate of 2 deg·min^−1^. Quantitative phase analysis was carried out by the corundum number method using the MDI JADE program.

## 3. Results

### 3.1. Rheological Studies

To enable the processing of the composite mixtures by injection molding, polypropylene was used as the matrix. The melt behavior was studied at high shear rates corresponding to the operating conditions of the injection-molding machine at a processing temperature of 210 °C.

The rheological characteristics of the melt were critically important for the injection-molding process, as they determined each material’s ability to evenly fill the mold, the quality of the final product, and the stability of the technological process. Optimal viscosity ensured uniform melt flow through the mold channels, preventing defects such as incomplete filling, air entrapment, and the phase separation of the mixture components. Polymer melts typically exhibit a viscosity dependence on shear rate; as the shear rate increases, viscosity decreases, which is particularly relevant for injection-molding processes.

**Figure 2:**
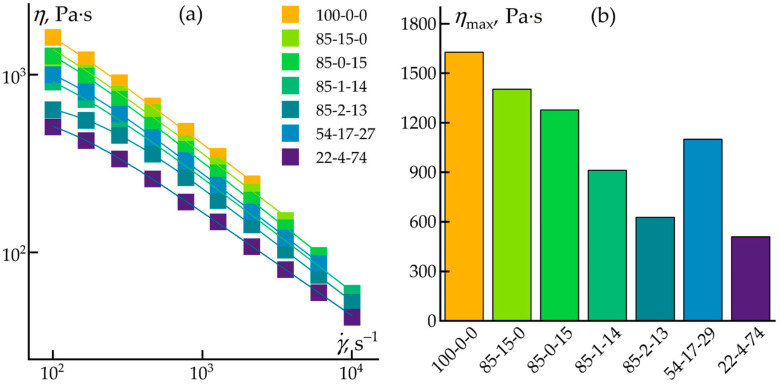
Flow curves (**a**) and maximum viscosities (**b**) of the investigated mixtures at 210 °C.

At the initial stage of this study, a capillary rheometer was used to determine the rheological properties of multicomponent mixtures over a wide range of shear rates. Figure 2 shows that all studied systems exhibited pseudoplastic behavior.

At a shear rate of 100 s⁻^1^, the viscosity was on the order of 10^3^ Pa·s, while an increase in the shear rate to 10^4^ s⁻^1^ resulted in an approximately 50-fold decrease in the viscosity. This reduction may be attributed to a weakening of the interfacial boundary between the polypropylene matrix and the added fillers, facilitating layer-by-layer sliding under the shear. The influence of the mixture composition on the melt flow is evident from the maximum viscosity values presented in Figure 2b. Experimentally, it was determined that three main parameters affected the composite’s viscosity: the polypropylene concentration, the polypropylene-to-cellulose ratio, and the fraction of the ES-40 additive. A decrease in polypropylene content would lead to lower viscosity, likely due to the presence of silica particles or oligomers acting as plasticizers. At high cellulose concentrations, the cellulose would begin to serve a structuring function, as evidenced by the formation of interwoven fibers—observed, for example, in the system with a 54-17-27 ratio, where cellulose constituted one-third of the matrix volume.

Despite the compositional variations, the influence of the components on the melt’s rheological properties within the shear rate range typical for injection molding was found to be negligible. This permits the use of these compositions without the need for additional equipment adjustments or extra plasticizers.

### 3.2. Molding of Specimens and Their Properties

**Figure 3:**
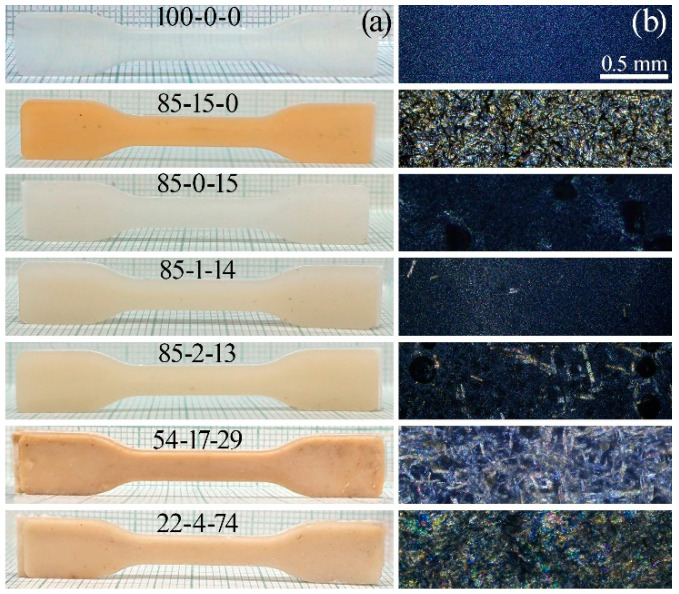
Images of the molded samples (**a**) and morphologies of extrudate films under crossed polarizers (**b**).

All the investigated compositions proved suitable for processing by injection molding and pressing, which enabled the production of samples for further investigation of their mechanical properties and morphology. Images of the samples are presented in Figure 3.

Visual inspection revealed that all samples exhibited homogeneity without any obvious defects such as cracks, delamination, or porosity. The addition of cellulose imparted a brown tint to the products, the intensity of which increased with the cellulose concentration; this is likely associated with the thermal processes that occurred during processing. In contrast, the incorporation of the ES-40 imparted an opalescent, translucent white coloration to the material.

The morphology of the samples was examined by optical microscopy (Figure 3b). Analysis showed that the neat PP exhibited birefringence under crossed polarizers due to its crystallinity. The addition of the ES-40 slightly reduced this effect, presumably due to the partial destabilization of the crystalline structure of the polypropylene.

Introducing cellulose at low concentrations (1%) had little impact on the microstructure, as the fibers were dispersed discretely and did not form an interconnected network. However, increasing the cellulose concentration to 2% resulted in a significant increase in the number of fibers, although they remained isolated and did not form a three-dimensional network. The most notable changes were observed in systems with high cellulose contents; at 17% cellulose, a three-dimensional network was clearly observed under the microscope. In compositions containing 4% cellulose and an excess of ES-40 (74%), interwoven fibers were detected, leading to the formation of a robust spatial structure.

**Figure 4:**
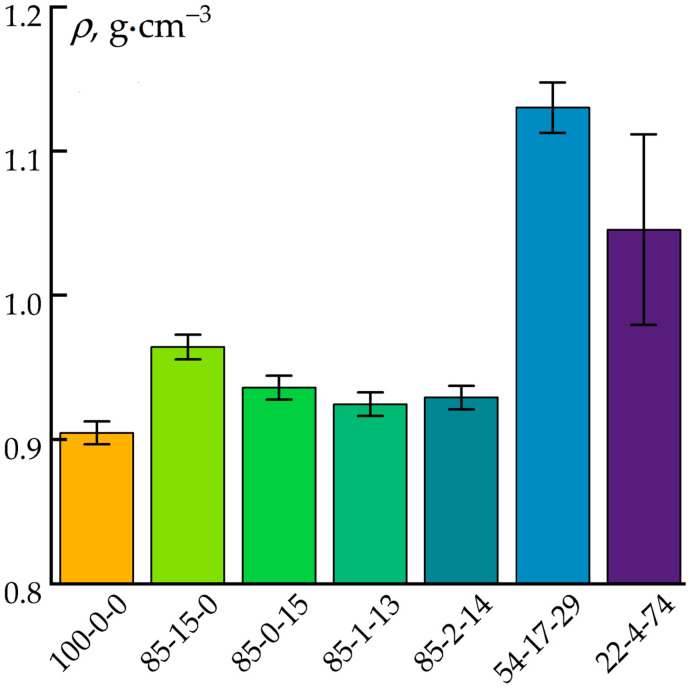
Densities of the obtained impellers.

The density of the composite products was a key parameter that determined both the weight and the operational performance of each final product. Achieving an optimal balance between density and strength is critical for creating materials that are both lightweight and durable. In this context, the influences of the cellulose and ES-40 content on the densities of the molded products were investigated. The results obtained are presented in Figure 4.

The incorporation of the cellulose and ES-40 into the PP matrix slightly increased the material’s density. Even in composites with high PP content (85%), the densities remained below 1 g·cm^−3^, which makes these composites attractive for lightweight structural applications. Interestingly, the addition of the pure cellulose (ρ = 1.6 g·cm^−3^) caused a more significant increase in density compared to the ES-40, which, during the process of hydrolytic polycondensation, converted to silica (SiO_2_) with a density of 2.2–2.7 g·cm^−3^. This difference may be related to the formation of a porous structure during the hydrolysis of the ES-40 in the mixing process. The formation of pores can adversely affect the mechanical properties of products by reducing their strength, which is especially critical for load-bearing applications.

**Figure 5:**
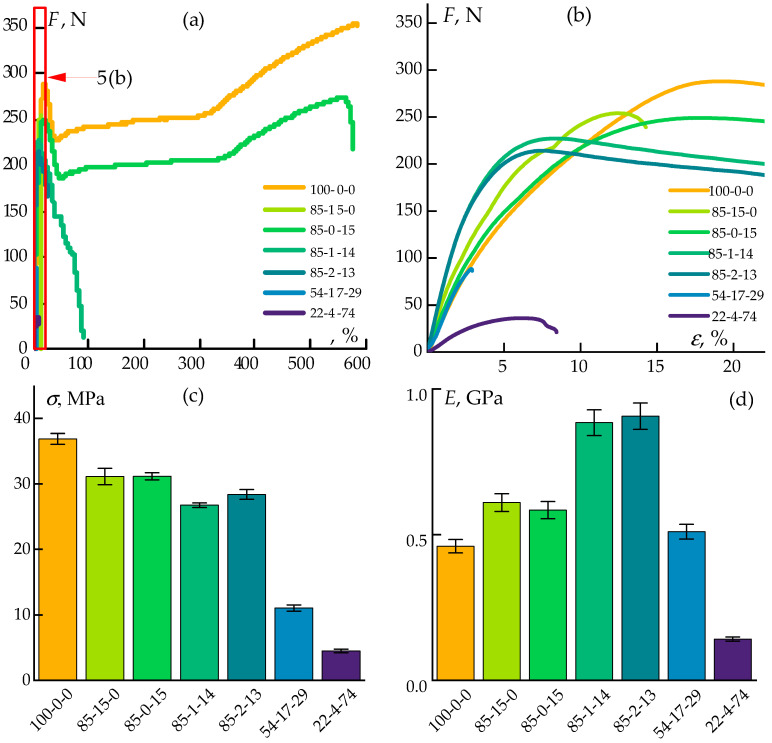
Deformation curves (**a**), zoomed areas of up to 20% (**b**), tensile strength (**c**), and tensile moduli (**d**) of the obtained samples.

Consequently, the mechanical properties of the obtained samples were investigated. The tensile test results are presented in Figure 5.

The inclusion of dispersed particles significantly affected the fracture behavior under tensile loading. Neat polypropylene failed by the necking mechanism (Figure 5a). The addition of 15% ES-40 reduced the stress during deformation but did not alter the failure mode.

In contrast, the incorporation of cellulose led to a change in the deformation behavior. The sample initially underwent neck formation and then failed. This behavior is attributed to the high concentration of cellulose fibers in the matrix, which hinders the orientation of polypropylene molecular chains, and to the low interfacial adhesion between the cellulose and the polymer matrix.

As the cellulose fraction increased, the elongation at break decreased, while the tensile strength at the onset of the necking remained nearly constant at approximately 30 MPa, which is slightly lower than that of neat PP (37 MPa). Both fillers enhanced the tensile moduli of the composites, with a particularly pronounced synergistic effect observed when small amounts of both cellulose and ES-40 were simultaneously introduced.

Nevertheless, the mechanical properties of the composites were primarily governed by the properties of the polymer matrix. As the PP concentration decreased, the tensile strength proportionally diminished. In particular, a sample with 22% polypropylene exhibited extremely low tensile strength and modulus values: a result of the compromised matrix integrity. Such a material is of particular interest for further carbonization aimed at producing bulk carbon–silicon carbide composites.

**Figure 6:**
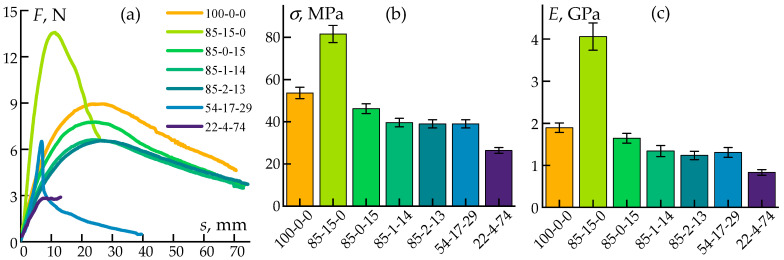
Deformation curves (**a**), flexural strength (**b**), and modulus (**c**) values.

Data on the flexural properties of the composites are presented in Figure 6.

Bending tests revealed reductions in deflection for the samples with the cellulose addition, although this effect was observed only at high fiber concentrations. At the same time, the flexural modulus and ultimate flexural strength doubled, but only for the highly cellulose-filled system (85-15-0).

Thus, it was established that the introduction of silica in the form of the ES-40 precursor does not improve mechanical properties. Meanwhile, the addition of cellulose at concentrations sufficient to form fiber entanglements in the composites contributed to a twofold increase in flexural strength and stiffness but also led to a twofold decrease in tensile strength. It is important to note that in all studied concentration ranges, the introduction of components allowed for the production of compositions with sufficient mechanical properties for processing into filaments suitable for 3D printing using the FDM method. This opens up prospects for the application of these materials in additive manufacturing.

### 3.3. Microcrystalline Cellulose

**Figure 7:**
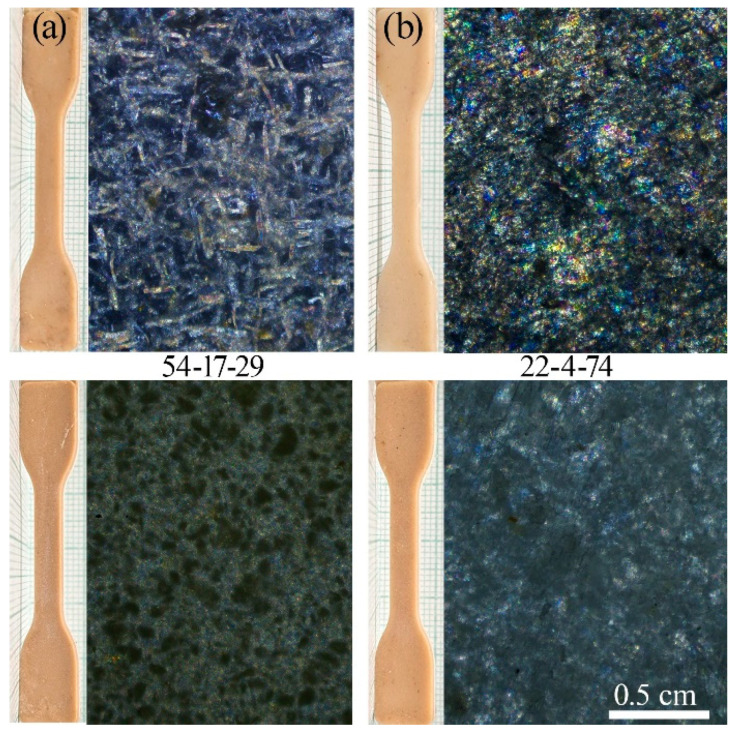
Photographs of samples and morphologies of extrudate films under crossed polarizers: 54-17-29 (**a**) and 22-4-74 (**b**).

A particularly interesting aspect was the comparison of microcrystalline cellulose with conventional fibrous cellulose. To investigate this, highly filled compositions with component (PP-cellulose-ES-40) contents of 54-17-29 and 22-4-74 were prepared, and their morphology, density, and mechanical properties were analyzed. Figure 7 presents photographs of the samples and a comparative analysis of the composite morphologies in the crossed polarizers.

The substitution of fibrous cellulose with microcrystalline cellulose (MCC) resulted in a significant change in the materials’ morphology. The composites became more homogeneous, without the pronounced birefringence of individual fibers, which can be attributed to the considerably smaller crystallite sizes of the MCC. The densities of the samples with the MCC increased in both series—from 1.05 to 1.10 g·cm^−3^ for the 22-4-74 series and from 1.10 to 1.15 g·cm^−3^ for the 54-17-29 series—indicating the formation of more monolithic composites with reduced porosity. Moreover, the enhanced structural homogeneity suggests improved bioavailability, as smaller particles are more susceptible to biodegradation.

**Figure 8:**
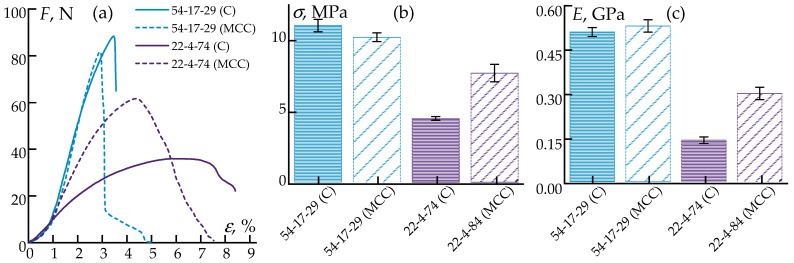
Deformation curves (**a**), tensile strengths (**b**), and tensile moduli (**c**) of the samples.

Comparative data on the tensile mechanical properties are presented in Figure 8.

The introduction of the MCC virtually did not alter the tensile properties of the compositions with high cellulose contents, whereas it led to a twofold increase in strength in the systems with low polypropylene contents. This can be attributed to the greater structural homogeneity achieved with the MCC. The load–strain curves indicate that the composites with MCC exhibited reduced elongation at break under maximum tensile stress.

**Figure 9:**
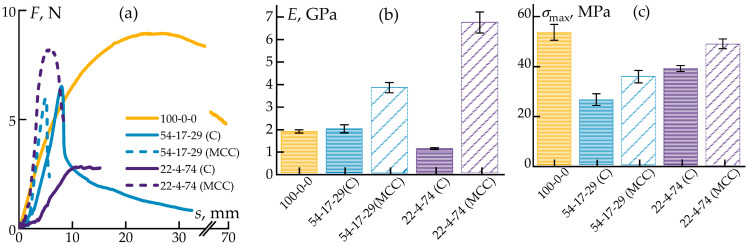
Deformation curves from three-point bending (**a**), along with the dependencies of the ultimate flexural stress (**b**) and flexural moduli (**c**) of the samples.

Figure 9 presents the deformation curves under bending.

Analysis revealed that MCC primarily influenced the flexural modulus, increasing it up to threefold in the systems with the lowest polypropylene fractions. In the presence of MCC, the ultimate flexural stress also increased, approaching the values of neat polypropylene even for highly filled composites containing only 22% PP. However, in all cases, significant reductions in the maximum flexural strain were observed.

Thus, the incorporation of the MCC raised the tensile strength to approximately 65 MPa and the Young modulus to about 4.2 GPa, values that are comparable with or exceed those of commonly used 3D-printing polymers such as PLA (50–70 MPa; 2.5–3.5 GPa [79]), ABS (30–45 MPa; 1.8–2.5 GPa [80]), and PETG (45–55 MPa; 2.0–2.3 GPa [81]), improving the mechanical properties of the composites compared to fibrous cellulose. The obtained results confirm that these polymer compositions not only can be effectively employed in injection-molded products but also have potential for the fabrication of filaments for 3D printing using composite plastics. Their use is particularly promising in applications requiring high stiffness, as well as for the production of precursor three-dimensional preforms for subsequent carbonization.

### 3.4. Carbonization

**Figure 10:**
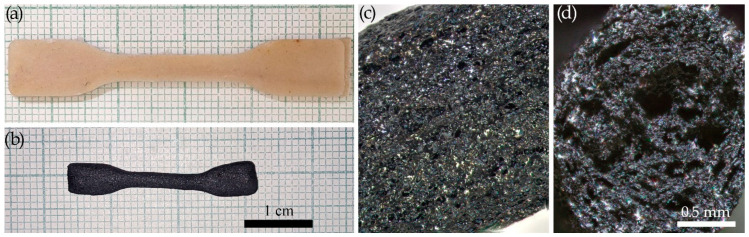
Comparison of the shape of the original impeller (**a**) and the sample after pyrolysis (**b**). Surface morphology (**c**) of the impeller and the cross-sectional fracture (**d**) observed by optical microscopy.

Particular interest was devoted to the possibility of utilizing the injection-molded composites as precursors for three-dimensional carbon–silicon carbide products. To test this hypothesis, preliminary pyrolysis experiments were conducted using the previous molded samples. The results are presented in Figure 10.

**Figure 11:**
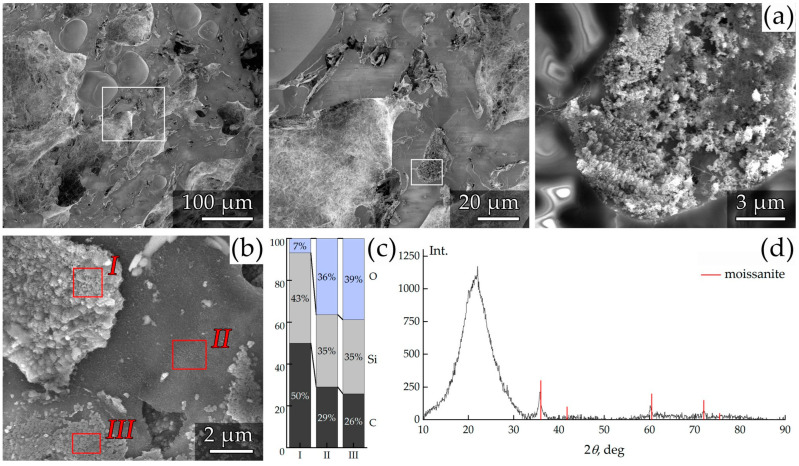
SEM images of the fracture morphology of the sample (**a**,**b**), along with compositional data obtained by EDS (**c**) and XRD (**d**) analysis.

This experiment demonstrated that after pyrolysis, the samples retained their overall shape while uniformly shrinking in all directions, although with some rounding of the edges. This confirms the feasibility of forming bulk thermally stable products with predetermined geometries. The density measurements revealed that the resulting samples possessed porous structures with a density of 1.27 g·cm^−3^ each. This finding was confirmed by the fracture morphology studies conducted using both optical and scanning electron microscopy (Figure 10 and Figure 11).

Microstructural analysis revealed a high porosity within the composite with a well-developed internal pore surface. Energy-dispersive X-ray spectroscopy (EDS) data (Figure 11) and X-ray diffraction (XRD) analysis established that the major portion of the solid inclusions consisted of silicon carbide particles corresponding to the crystalline structure of moissanite. According to the compositional analysis, the matrix of the material primarily comprised an amorphous phase of silica with carbon.

## 4. Conclusions

In this study, the properties of composites based on a polypropylene matrix containing fibrous or microcrystalline cellulose and a silica precursor in the form of ES-40 were investigated with the aim of enhancing their bioavailability and suitability for additive manufacturing applications.

Rheological analysis demonstrated that the developed multicomponent systems exhibited pseudoplastic behavior, with a reduction in viscosity over the shear rate range typical for injection-molding operations. Morphology analysis and density measurements revealed that the addition of cellulose promoted the formation of a three-dimensional network within the matrix, which positively influenced the flexural mechanical properties—resulting in a two- to threefold increase in the flexural modulus. In contrast, the addition of cellulose led to a reduction in tensile strength, attributed to limited interfacial adhesion and hindered orientation of the polypropylene molecular chains. The substitution of fibrous cellulose with microcrystalline cellulose improved the composite’s homogeneity, increased its density, and enhanced its mechanical properties, particularly in systems with low polypropylene contents.

Preliminary pyrolysis experiments demonstrated that the injection-molded composites can serve as precursors for the fabrication of three-dimensional carbon–silicon carbide products with low density. After pyrolysis, each product retained its geometry with uniform shrinkage and rounded edges. The resulting well-developed porous structures, as confirmed by optical and scanning electron microscopy along with EDS and XRD analyses, indicate the formation of silicon carbide particles corresponding to the crystalline phase of moissanite embedded in a composite matrix of silica and carbon.

Although the PP matrix is not biodegradable itself, the incorporation of microcrystalline cellulose, a renewable and partially degradable filler, will improve the environmental profiles of composites. Using ES-40, an inorganic precursor, will avoid the use of toxic additives and create stiff PP-MCC composite materials, which can lead to the formation of stable ceramic residues during pyrolysis.

## Figures and Tables

**Table 1:** Compositions of the investigated mixtures.

Sample Abbreviation	Component Concentration, wt.%		
	Polypropylene	Cellulose	ES-40
100-0-0	100	0	0
85-15-0	85	15	0
85-0-15	85	0	15
85-1-14	85	0.8	14.2
85-2-13	85	1.5	13.5
22-4-74	22.1	3.9	74
54-17-29	54	17	29

## Data Availability

Data are contained within the article.

## Abbreviations

The following abbreviations are used in this manuscript:

FDM	Fused Deposition Modeling
MCC	Microcrystalline cellulose
SiC	Silicon carbide
PAN	Polyacrylonitrile
DOAJ	Directory of Open-Access Journals
PIM	Powder injection molding
ES-40	Ethyl silicate 40
SEM	Scanning electron microscopy
EDS	Energy-dispersive X-ray spectroscopy
XRD	X-ray diffraction
